# Estimating the Incidence of Typhoid Fever and Other Febrile Illnesses in Developing Countries

**DOI:** 10.3201/eid0905.020428

**Published:** 2003-05

**Authors:** John A. Crump, Fouad G. Youssef, Stephen P. Luby, Momtaz O. Wasfy, Josefa M. Rangel, Maha Taalat, Said A. Oun, Frank J. Mahoney

**Affiliations:** * Centers for Disease Control and Prevention, Atlanta, Georgia, USA; † U.S. Naval Medical Research Unit No. 3, Cairo, Egypt; ‡ Ministry of Health and Population, Cairo, Egypt

**Keywords:** Population surveillance, community surveys, incidence, fever, typhoid fever, *Salmonella* Typhi, brucellosis, *Brucella*, malaria, Egypt, research

## Abstract

To measure the incidence of typhoid fever and other febrile illnesses in Bilbeis District, Egypt, we conducted a household survey to determine patterns of health seeking among persons with fever. Then we established surveillance for 4 months among a representative sample of health providers who saw febrile patients. Health providers collected epidemiologic information and blood (for culture and serologic testing) from eligible patients. After adjusting for the provider sampling scheme, test sensitivity, and seasonality, we estimated that the incidence of typhoid fever was 13/100,000 persons per year and the incidence of brucellosis was 18/100,000 persons per year in the district. This surveillance tool could have wide applications for surveillance for febrile illness in developing countries.

Measuring the incidence of febrile illness caused by various pathogens poses a major public health challenge because hospital-based approaches capture only a fraction of patients, clinical diagnosis is usually unreliable, and diagnostic tests are often not available in disease-endemic countries (*1*). Consequently, the incidence and relative importance of the etiologic agents of the febrile illness remain unknown in many parts of the world. Public health personnel have insufficient data to make the disease burden (incidence, illness, and death) estimates to guide priorities for the use of scarce health resources (*2*) and to help refine policy on the empiric management of febrile patients (*3*).

Attempts to measure the incidence of febrile illness have been hampered by problems associated with surveillance sensitivity and surveillance specificity. Sensitivity is determined largely by the placement of the surveillance system within the healthcare system and the completeness of enrollment of case-patients (Figure). Although conducting surveillance at the tertiary hospital level is attractive from the perspective of laboratory capacity and infrastructure, such surveillance captures only the most severe illnesses in persons who have access to hospital care. Hospital-based approaches tend to underestimate disease incidence. Routine door-to-door visits to every household in a community to identify febrile persons and then collect diagnostic specimens is highly sensitive but limited by cost and time considerations (*4*). Specificity is determined largely by the diagnostic criteria used in the surveillance case definition. Syndrome-based surveillance requires no laboratory capacity but lacks specificity because the causes of febrile illnesses may be clinically indistinguishable. Therefore, syndrome-based surveillance frequently results in classification errors. To maximize specificity, the case definition for the febrile illnesses under surveillance must include a positive result from a reliable diagnostic test.

**Figure:**
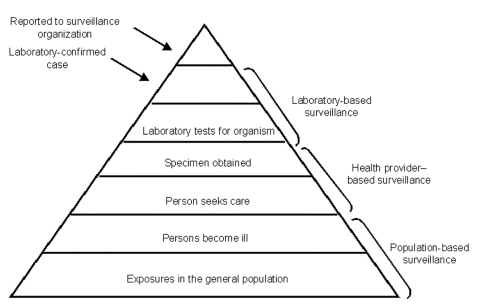
Febrile illness surveillance pyramid.

A sensitive and specific surveillance system that accurately measures the incidence and causes of febrile illness in a country or region must be able to detect cases as close as possible to the population level (Figure) and must be supported by modern laboratory diagnostic capacity. Because such surveillance is labor-intensive and expensive, a rapid method that measures incidence and cause in sentinel populations of a country or region over a finite period of time is needed. Such sentinel surveillance could be repeated at intervals to detect changing patterns of disease.

We developed a rapid sentinel surveillance tool to determine the causes and measure the incidence of febrile illness. We pilot-tested this tool in Egypt, where the Egypt Ministry of Health and Population and the U. S. Naval Medical Research Unit No. 3 (NAMRU-3) have recently collaborated to expand laboratory capacity at district fever hospitals as part of a plan to strengthen national hospital-based surveillance for febrile illnesses. Fever hospitals are tertiary referral centers for persons with suspected infectious disease. By using our surveillance tool in concert with Egypt’s expanded laboratory capacity, we aimed to determine the etiologic agent and to measure the incidence of the leading causes of prolonged fever in Bilbeis District.

## Methods

### Study Site

Bilbeis District, Sharkia Governorate, Lower Egypt, was chosen as the study site. Bilbeis District has a population of 664,000 and comprises a rural hinterland and centrally located Bilbeis City. Rural Bilbeis District comprises scattered villages and hamlets that rely largely on subsistence agriculture. Bilbeis City consists of high-density single- and multiple-story dwellings. The relatively close proximity of this district to Cairo provided practical advantages for epidemiologic and laboratory support.

### Household Survey

We conducted a household survey during August 2000 and January 2001 in Bilbeis and neighboring Fakkous Districts, Lower Egypt. The survey was part of a larger study that evaluated injection practices in several parts of Egypt (*5*). Our goal was to determine patterns of health-seeking behavior among persons reporting prolonged fever (self-diagnosed fever >3 days’ duration) in Bilbeis and Fakkous Districts during the 3-month period before the interview. The two districts were divided into 40 rural sites of approximately equal population. Four of the sites were randomly selected. In these four rural sites, a census was conducted of all households and household members. The study team spent 1 week working in each rural site. All persons living in each household were invited to participate in the interview by answering a structured questionnaire. If a household member was absent on the day of the visit, the study team returned at least once during the 1-week period. For children <10 years of age, the head of the household was interviewed.

### Sentinel Surveillance

We obtained a contemporary census of all district health services and health providers from the Bilbeis District Health Office and used the findings of the household survey to identify categories of health providers who were seeing patients with prolonged fever. We used data from the census of district health services and health providers as the sampling frame from which to select health providers for the febrile illness surveillance system. One district fever hospital, 11 fever specialists, and 68 primary care providers (general practitioners, internal medicine physicians, pediatricians, and rural health unit doctors) were recorded in the contemporary health services and health provider census of the Bilbeis District Health Office. We conducted the surveillance from July through October 2001 at the fever hospital, among the 11 district fever specialists, and among a random selection of 10 (15%) of 68 other representative health providers.

During the 4-month study period, all persons of ages >6 months who visited a surveillance health provider in Bilbeis District with current fever of >3 days’ duration were invited to participate. After obtaining informed consent from these febrile patients, health providers administered a brief questionnaire that captured demographic and clinical information; blood was collected for culture and serologic testing. Health providers were given a small financial incentive to compensate for the additional time required to enroll patients. Persons <6 months of age were not included because this group is understood to be at low risk for typhoid fever, in part because of predominant or exclusive breastfeeding (*4*). Ethical approval was obtained from the Institutional Review Boards of NAMRU-3 and the Centers for Disease Control and Prevention.

### Laboratory Capacity and Methods

We trained healthcare providers on obtaining blood culture, sterile technique, and needle safety and supplied these providers with materials for venipuncture and blood culture. Couriers visited each provider every day to ensure adequate laboratory supplies and transport laboratory samples and test results. Microbiology laboratory technicians at the Bilbeis fever hospital were trained in blood culture technology and retrained in basic bacteriology. The medical microbiology laboratory was equipped with biological safety equipment, an incubator, and other materials necessary to process blood cultures and identify bacteria. The NAMRU-3 bacteriology laboratory in Cairo provided training, quality control on all samples and bacterial isolates, a reference laboratory, and special test capacity.

The Phase2 bi-phasic blood culture system (PML Microbiologicals, Wilsonville, OR) was used. We incubated bottles for 14 days at 35°C and observed them daily for signs of microbial growth. Growth in broth or on agar paddles was examined by Gram stain and was subcultured to solid media for identification. Serologic testing for *Brucella* spp. was performed by standard tube agglutination with *Brucella abortus* antigen (SA Scientific, San Antonio, TX).

### Incidence Calculations

Incidence was calculated for each examined disease after accounting for the provider-sampling scheme, test sensitivity and specificity, and seasonality. Multipliers to account for the provider-sampling scheme were derived arithmetically by using the provider populations sampled in our study as the numerator and the provider populations known from the contemporary census of all district health services and health providers as the denominator. Because the sentinel surveillance system included the only district fever hospital and all district fever specialists, no multiplier was applied to cases detected at these sites. However, to account for sampling, only 15% of primary health providers in the district, a multiplier of 6.8 was applied to cases detected among primary healthcare providers.

Multipliers to account for shortcomings of test sensitivity were derived by reviewing published systematic studies of the performance of the diagnosed tests used in our study compared with standard criterion tests. The sensitivity of a single blood culture for the diagnosis of typhoid fever has been estimated as 50% when compared with bone marrow aspirate culture (*6*); therefore, a multiplier of 2.0 was applied to account for blood culture–negative typhoid fever. Because most brucellosis cases can be detected with the combination of blood culture and the tube agglutination assay, no multiplier was applied to account for test sensitivity for brucellosis. For the purposes of incidence calculations, test specificity for typhoid fever and brucellosis was assumed to approach 100%.

Multipliers to account for seasonal variation in disease incidence were derived from syndrome- and laboratory-based febrile illness surveillance systems. Because approximately 45% of typhoid fever occurs from June through October in Egypt (national syndrome-based surveillance system for typhoid fever, unpub. 2000), a multiplier of 2.2 was applied to adjust for the whole year. A similar seasonal pattern occurs for brucellosis (national acute febrile illness surveillance system, unpub. data, 2000) (Table).

**Table:** Incidence estimates for typhoid fever and brucellosis, Bilbeis District, Egypt, 2001

Disease	No. of cases captured by surveillance site type Crude (adjusted^a^)				Test sensitivity multiplier	Seasonality multiplier	Total cases	Incidence (/100,000)
	Fever hospital)	Fever specialist	Primary provider	Total				
Typhoid fever	6.0 (6.0)	13.0 (13.0)	0.0	19.0 (19.0)	2.0	2.2	83.6	12.6
Brucellosis	15.0 (15.0)	12.0 (12.0)	4.0 (27.2)	31.0 (54.2)	1.0	2.2	119.2	18.0

^a^Adjusted for health provider sampling scheme. No multiplier is applied for cases identified at the fever hospital and among fever specialists. A multiplier of 6.8 is applied for cases identified among primary providers.

### Statistical Methods

Data were stored and analyzed with Epi Info version 6.04 (Centers for Disease and Prevention, Atlanta, GA). Incidence calculations were made by using Microsoft Excel 2000 (Microsoft Corp. Redmond, WA) spreadsheets.

## Results

### Household Survey

A census of the four randomly selected rural sites recorded 369 households. Interviews were completed in all 369 randomly selected households. Of the 2,421 persons eligible for interview in survey households, 363 (15.0%) could not be interviewed because the participant was absent during the 1-week survey period. No eligible person refused to be interviewed. In total, 2,058 (85.0%) of 2,421 eligible persons, or their guardians, were interviewed. Of persons interviewed, 474 (23.0%) reported having fever of >3 days’ duration (i.e., prolonged fever) during the previous 3 months. Of those reporting prolonged fever, 379 (80.0%) sought care from a health provider. Of those seeking care from a health provider, 340 (89.7%) saw a physician, 32 (8.4%) saw a pharmacist, and 7 (1.8%) saw a layperson. Of the 340 who saw physicians, 274 (80.6%) saw a private physician, 36 (10.6%) saw a rural health unit physician, 19 (5.6%) saw a physician at a district general hospital, 7 (2.1%) saw a physician working for a health insurance organization, and 1 (0.3%) saw a physician at the district fever hospital.

### Sentinel Surveillance

In total, 449 patients with prolonged fever were enrolled at the sentinel surveillance sites. No eligible patients refused to participate. *Salmonella* *enterica* serotype Typhi (*Salmonella* Typhi) was isolated by blood culture from 19 (4.2%) patients. The median age of patients with typhoid fever was 22 years (range 5–60 years), and 5 (26.3%) patients were female. *Brucella* spp. were isolated by blood culture from 15 (3.3%) patients, and brucellosis was confirmed by positive tube agglutination assay (titer of >1:160) for another 16 (3.6%). The median age of patients with brucellosis was 31 years (range 11–60 years); 12 (38.7%) patients were female. *Escherichia coli* and *Hemophilus influenzae* serotype b were each isolated by blood culture from one patient. No non-Typhi *Salmonella* serotypes were isolated.

The contamination of blood cultures with skin flora (e.g., coagulase-negative *Staphylococcus*, diphtheroids), resulting from poor sterile technique, was a problem during the early part of the study. Active monitoring and intensive retraining of participating health providers reduced the blood culture contamination rate from 15% during the first 2 months of the study to 7% during the second 2 months of the study (p<0.01).

Of patients with brucellosis, 26 (87.1%) of 31 were diagnosed with and treated clinically for typhoid fever. In total, 302 (71%) of 423 patients were already using an antimicrobial agent at the time they sought treatment by a health provider participating in the sentinel surveillance system. Patients most frequently reported taking amoxicillin and chloramphenicol. However, no significant association was found between current antimicrobial therapy and yield of pathogens by blood culture.

### Incidence Calculations

After we made adjustments to account for the provider-sampling scheme, test sensitivity, and seasonality, we estimated the annual typhoid incidence rate as 13/100,000 persons and the annual brucellosis incidence rate as 18/100,000 persons. The multipliers and calculations used to derive these estimates are summarized in the Table.

## Discussion

Before our study, the most reliable existing estimates of the typhoid fever incidence rates in Egypt were established during typhoid vaccine studies conducted more than two decades earlier. These studies documented an annual typhoid fever incidence of 209/100,000 persons in 1972–1973 (*7*) and of 48/100,000 persons in 1978–1981 (*8*,*9*) among school-aged children in Alexandria, Egypt. Vaccine studies may overestimate typhoid fever incidence because they may be preferentially conducted in areas of known high incidence of typhoid and in groups at high risk of acquiring typhoid fever (e.g., school-aged children). Our study, conducted among all age groups in a single district, showed annual typhoid fever incidence rates that were lower, at 13/100,000 persons. This finding is consistent with a study design that was not targeted to a high incidence population. A lower typhoid fever incidence may also be consistent with reductions of other enteric diseases reported in Egypt, resulting from improved management of diarrheal disease (*10*) and the large and growing proportion of persons living in both rural and urban areas who have access to safe water (*11*).

Our study demonstrated that brucellosis was as important as typhoid fever as a cause of prolonged fever in Bilbeis District. Brucellosis has increasingly become recognized as a public health problem in Egypt, as it has in Kuwait and other countries in the Middle East. Our estimated annual rate of 18/100,000 persons approaches that found in Kuwait during the 1980s (*12*). Because brucellosis and typhoid fever have similar signs and symptoms, brucellosis frequently was misdiagnosed as typhoid fever, resulting in provision of inadequate antimicrobial therapy.

Healthcare provider–based surveillance previously has been used to capture typhoid fever cases for vaccine studies (*13*) and to measure typhoid fever incidence (*14*). However, such an approach requires that febrile persons report to the health providers participating in the study (Figure). We conducted a household survey to assess patterns of health-seeking behavior in Bilbeis District before implementing surveillance. We improved the efficiency of this step by integrating questions of health-seeking behavior for febrile persons with an existing population-based survey (*5*). The reliability of the household survey data could be improved by following classic cluster sampling methods (*15*,*16*). One way to achieve this would be integration with national demographic and health surveys that use cluster-sampling methods and are conducted at regular intervals in many developing countries.

Identifying, assessing, and strengthening a central laboratory capacity is a key foundational step for implementing our surveillance tool. Our sentinel surveillance study was built on recently expanded laboratory capacity within Egypt’s district fever hospitals. Other investigators have successfully identified, assessed, and strengthened central laboratory capacity for sentinel hospital-based studies of febrile illness (*17*–*19*). We extended capacity beyond the tertiary hospital and into the community to determine the etiologic agents and to estimate the incidence of febrile illness closer to the population level.

Several factors must be considered when assessing the accuracy of this surveillance tool for measuring the incidence of febrile illnesses. The febrile illness surveillance tool may not capture mild disease. Although mild illness does not contribute substantially to disease burden, using a broader case definition for both the household survey and for the surveillance system might have captured more cases. For typhoid fever in particular, a broader case definition might have captured more cases among children <5 years of age who may experience milder illness (*4*) than adults. In addition, including infants <6 months of age would be important for measuring the incidence of infectious diseases that, unlike typhoid fever, occur frequently in this age group.

That healthcare providers enroll all eligible patients and use sterile venipuncture technique are vital to the success of this tool. We maximized enrollment by using a financial incentive and controlled blood culture contamination by active monitoring, regular feedback, and retraining health providers. Although we reduced blood culture contamination to 7% during the second half of the study, the overgrowth of contaminants may have prevented us from recovering pathogens from a proportion of blood cultures.

Implementing our surveillance tool in countries with a larger informal healthcare sector (e.g., traditional healers, informal pharmacists) would present challenges. These challenges would include identifying, training, and maintaining the participation of all healthcare providers. Implementing this surveillance system would also be difficult where a larger proportion of the population lacks access to any healthcare. Community use of antimicrobial agents was high among patients enrolled in our study, reflecting the global epidemic of community antibiotic abuse (*20*). Use of an antimicrobial agent before venipuncture is known to reduce the sensitivity of blood culture in the diagnosis of typhoid fever (*6*) and other infectious diseases, although we were not able to demonstrate this reduction with our data. For typhoid fever, we used the lower rather than higher reported sensitivity in blood culture to adjust our crude disease rates to account for community antibiotic use (*6*,*21*). In the future, data might be available to develop multipliers for the impact of antimicrobial agent use on blood culture sensitivity for specific infections. Furthermore, the epidemiology of typhoid fever within a country is likely to be heterogeneous in both time and location. Febrile illness surveillance should be replicated in several representative districts before making inferences about national disease incidence.

Our febrile illness surveillance tool could be applied in other countries and regions and lends itself to periodic and rapid implementation in multiple sites. For example, such sentinel surveillance could be conducted every 5 years in a region to update disease incidence assessments and to guide syndrome-based patient management. Potential applications extend beyond typhoid fever surveillance. The surveillance tool may provide a solution to the difficulties of measuring disease incidence that are faced for many causes of febrile illness (*22*). For example, because of asymptomatic parasitemia, a febrile event can be reliably attributed to malaria only when other causes of fever are excluded, a luxury not afforded to primary healthcare providers in developing countries and seldom available even in clinical malaria studies. Our model serves as a platform, whereby conducting additional tests (e.g., thick and thin blood smears for malaria parasites, acute- and convalescent-phase serologic tests) might permit the simultaneous measurement of the incidence of a broad range of causes of febrile illness. In so doing, the fraction of febrile illness attributable to various etiologic agents can be estimated simultaneously for a country or region. Collecting additional diagnostic specimens (e.g., urine) would permit the assessment of relevant biomarkers (e.g., the level of community antibiotic use) (*23*,*24*). The model likely could be integrated with sentinel surveillance for diseases such as typhoid, brucellosis, leptospirosis, malaria, and melioidosis, and for a range of viral and rickettsial diseases. The resulting data could be used to guide appropriate local modifications of algorithms for the empiric management of febrile persons (e.g., the fever module of the World Health Organization/United Nations Children’s Fund guidelines for the integrated management of childhood diseases), especially in areas with low prevalence of malaria (*25*,*26*).

In Egypt, the febrile illness surveillance tool will be assessed in other districts to develop a national picture of the current incidence and causes of febrile illness. These data will help Egypt set priorities for spending for control measures and target specific prevention activities for a group of diseases that have thus far eluded accurate enumeration and standardized comparisons of incidence.
